# Supplementary material for: Hemodynamic Effects of Entry and Exit Tear Size in Aortic Dissection Evaluated with In Vitro Magnetic Resonance Imaging and Fluid-Structure Interaction Simulation
Source: ArXiv. 2023 Mar 23:arXiv:2303.13639v1. Preprint. [Version 1] (PMC10055490)
Supplement: 1 [file NIHPP2303.13639V1-supplement-1.pdf]

Supplementary Material

See below for supplementary figures and tables S1-S3, S9, S10. Upon publication we will make supplementary videos (.mp4) available. These are numbered S4-S8, with captions printed at the end of this document.

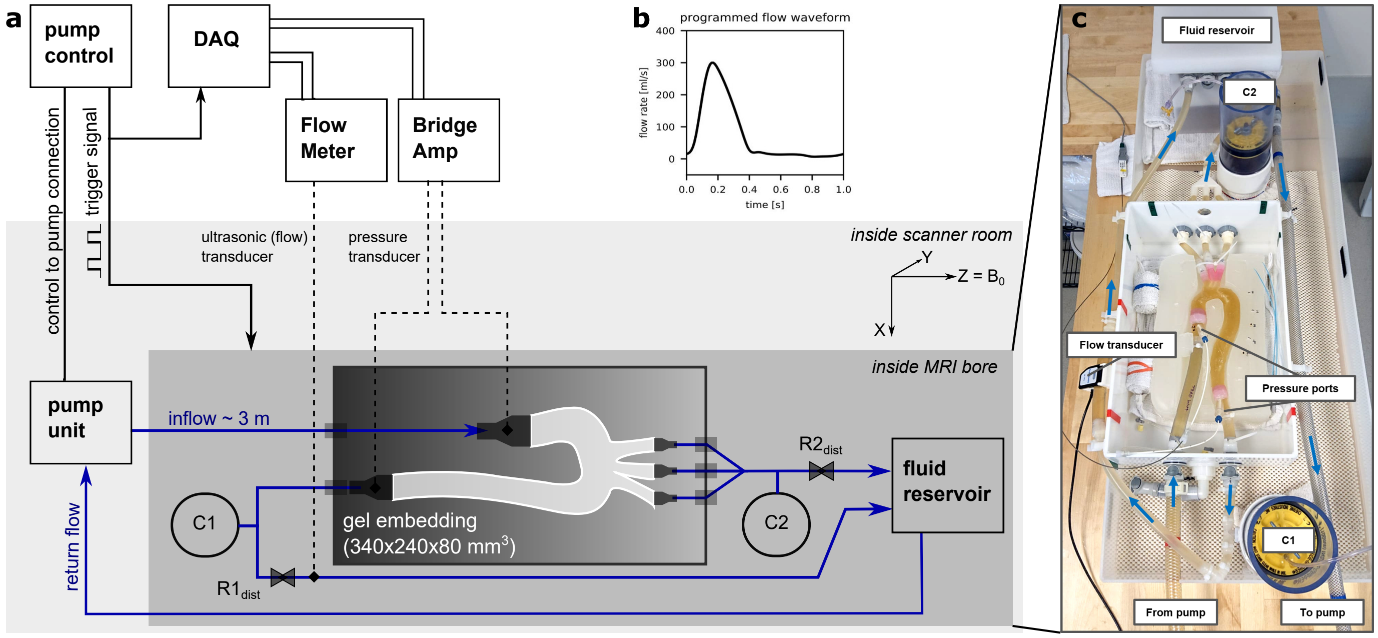

S1 Experimental setup. (a) Schematic drawing of the flow loop setup; fluid lines are displayed in blue. (b) Programmed flow rate waveform with stroke volume of 74.1 mL/s. (c) Photograph of the setup (with gel top and inner box lid both removed). All shown parts were inside the scanner bore during image acquisition. All other equipment (not shown in photograph) was positioned either outside the bore (pump unit), or outside the scanner room (pump control, data acquisition system (DAQ), bridge amplifier, flow meter).

	R_T (MPa s m^{-3})	C_T (m^3 Pa^{-1})	k_d (-)	k_s (MN m^{-3})	c_s (kN s m^{-3})	ρ_f (kg m^{-3})	μ_f (Pa s)	ρ_s (kg m^{-3})	$E_{y,t}$ (MPa)
TBAD$_{OR}$	150	6.74×10^{-9}	0.84	−18	−30	1100	0.0042	1450	1.2
TBAD$_{EN}$	161	1.23×10^{-8}	0.87	−18	−30	1100	0.0042	1450	1.2
TBAD$_{EX}$	170	1.02×10^{-8}	0.86	−18	−30	1100	0.0042	1450	1.2

S2. FSI simulations parameters. The three-element Windkessel boundary parameters include the total resistance R_T, total capacitance C_T, and ratio of distal to proximal resistance k_d. R_T and C_T are distributed across outlets according to measured flow splits (see Table 2 in main article) and the respective value for k_d. ETS scalar parameters k_s (elastic response) and c_s (viscoelastic response) were chosen to match the minimum-to-maximum dilation of the simulation to MRI-measured values. Fluid and structural density (ρ_f and ρ_s), fluid viscosity μ_f, and elastic modulus $E_{y,t}$ were prescribed according to benchtop measurements or manufacturer's information.

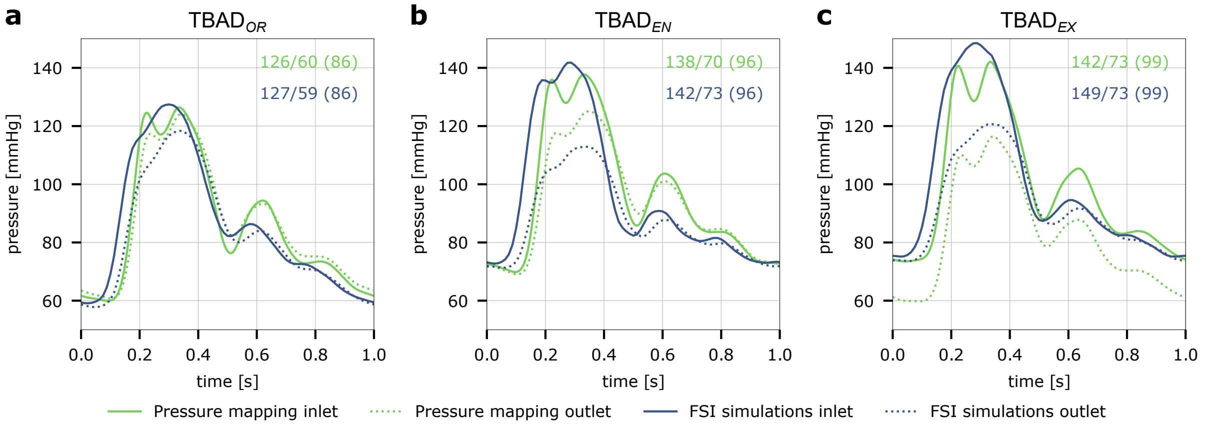

S3. Pressure waveforms at inlet (solid) and outlets (dotted) for catheter-based measurements (green) and FSI simulations (blue): (a) TBAD$_{OR}$, (b) TBAD$_{EN}$, and (c) TBAD$_{EX}$. Numbers in plotting areas report P_{sys}/P_{dias} (P_{MAP}) in mmHg.

	AAo	*TL1*	*TL2*	*TL3*	*TL4*	*TL5*	*FL1*	*FL2*	*FL3*	*FL4*	*FL5*	DAo_{dist}
**MRI (pulsatile flow, retrieved from 2D-cine data)**												
$TBAD_{OR}$												
A_0	612.4	339.1	182.0	118.8	73.5	62.3	517.4	587.7	540.3	551.5	569.5	444.7
A_{max}	633.9	345.9	185.7	121.7	74.5	63.2	530.9	606.2	553.5	572.1	590.9	453.9
A_{mean}	620.2	340.9	183.0	120.3	73.8	62.2	521.3	592.8	543.4	558.5	577.0	447.2
$TBAD_{EN}$												
A_0	630.3	253.7	148.9	97.3	83.7	57.2	490.5	489.6	475.1	477.0	450.2	398.5
A_{max}	656.9	262.6	155.0	99.8	85.7	57.5	496.6	499.8	486.4	493.6	466.1	411.5
A_{mean}	640.1	254.4	150.0	98.0	84.8	57.0	490.4	492.0	476.7	480.8	454.6	403.5
$TBAD_{EX}$												
A_0	559.0	272.2	151.9	95.0	77.6	54.3	428.5	466.2	456.2	477.1	464.8	382.9
A_{max}	581.6	274.8	152.9	95.9	78.0	55.5	444.1	481.6	470.3	500.1	482.9	389.1
A_{mean}	566.8	271.7	151.6	95.3	77.3	54.7	433.1	470.3	459.4	484.8	470.3	384.9
**MRI (steady flow, retrieved from 3D-SPGR data)**												
$TBAD_{OR}$	527.0	315.0	154.0	85.0	81.0	65.0	547.0	587.0	553.0	564.0	457.0	457.0
**MRI ("flow-off", retrieved from 3D-SPGR data)**												
$TBAD_{OR}$	435.0	268.0	152.0	85.0	83.0	60.0	418.0	431.0	411.0	438.0	445.0	373.0
**FSI (pulsatile flow)**												
$TBAD_{OR}$												
A_0	513.5	239.6	150.7	68.4	58.9	56.9	497.2	457.4	494.8	479.0	448.0	379.6
A_{max}	538.7	247.4	155.7	70.7	59.8	57.1	516.8	477.7	515.4	500.8	468.3	394.6
A_{mean}	523.2	242.2	152.1	69.1	59.1	56.9	505.2	465.5	502.9	487.5	455.9	385.5
$TBAD_{EN}$												
A_0	518.5	238.3	151.2	68.7	59.0	56.9	499.6	462.2	499.3	483.7	452.2	382.9
A_{max}	544.2	250.2	160.6	73.0	60.5	57.1	509.9	472.6	511.2	497.9	466.2	393.2
A_{mean}	526.7	241.5	153.5	69.8	59.4	57.0	503.4	466.1	503.9	489.0	457.4	386.8
$TBAD_{EX}$												
A_0	519.3	240.8	151.1	68.6	59.0	56.9	502.9	463.3	500.3	484.7	453.0	355.8
A_{max}	546.8	247.8	155.2	69.9	59.1	57.1	525.5	486.2	524.0	510.0	476.9	366.1
A_{mean}	528.5	243.1	152.2	68.9	58.9	56.5	510.6	471.2	508.3	493.2	461.0	359.7
**FSI (steady flow)**												
$TBAD_{OR}$	522.1	241.6	151.6	68.9	59.1	57.0	505.2	465.4	502.6	487.0	455.4	385.2
**STL model ("flow-off")**												
$TBAD_{OR}$	492.6	235.6	149.2	67.6	58.4	56.6	476.3	436.3	475.0	458.7	429.3	365.5

S9. Cross-sectional area (in mm^2) at twelve landmarks for pulsatile flow, steady flow, and "flow-off" modes. A_0 (area at first frame of the cardiac cycle) and A_{max} (maximum area of cardiac cycle) were considered to define end-diastolic and peak-systolic cross-sectional area, respectively. A_{mean} reports the area averaged over the cardiac cycle. For landmark label definition see Fig. 2a of main article. Refer to Supplementary Fig. S10 for data plots.

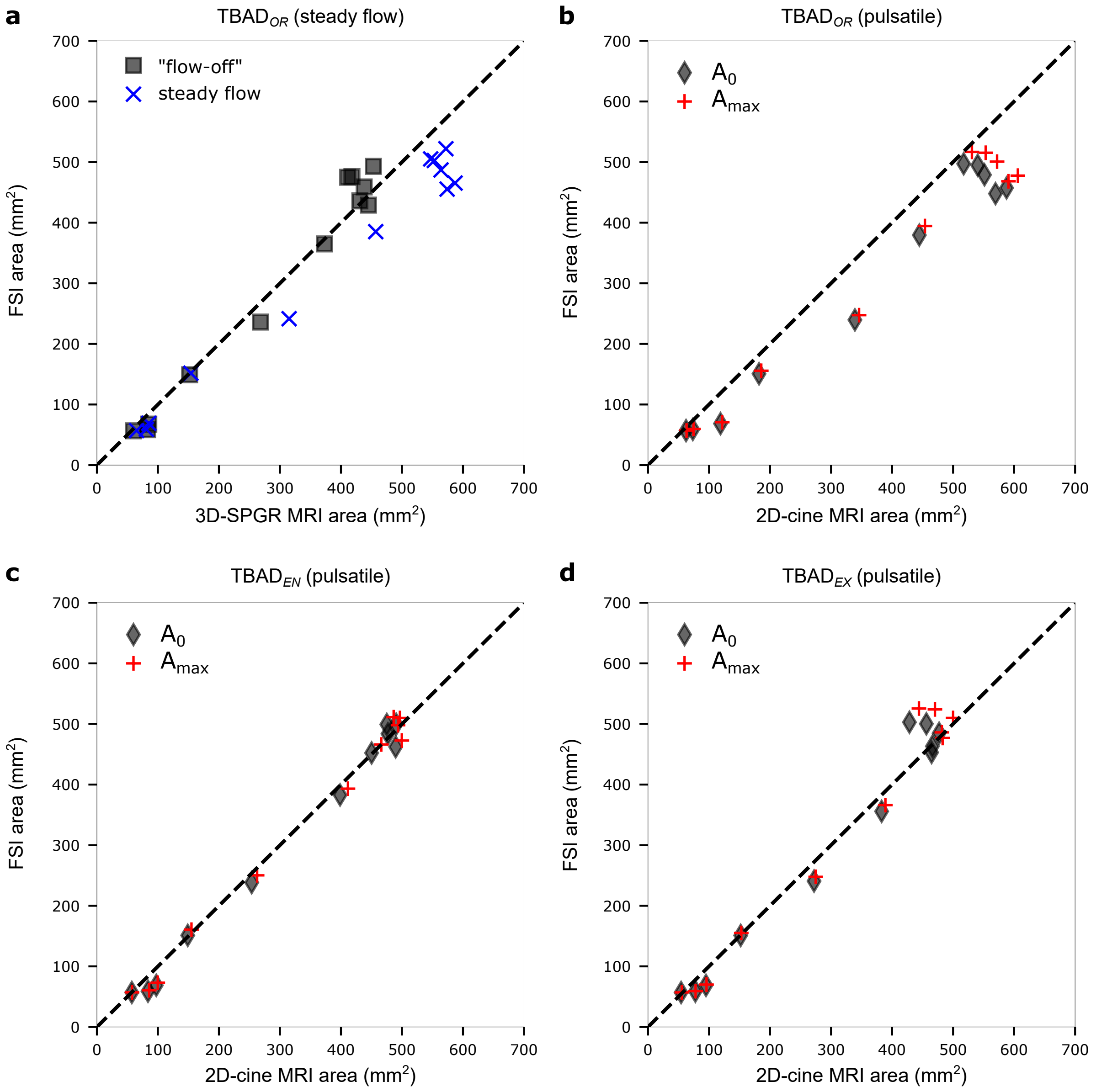

S10. Absolute cross-sectional area evaluated at twelve landmarks with 3D-SPGR or 2D-cine MRI of the 3D-printed model (horizontal axis) and based on the deformable structural domain, i.e. aortic wall, in FSI simulations (vertical axis): (a) “flow-off” and steady flow measurements; (b, c, d) first frame (A_0, end-diastolic) and maximum (A_{max}, peak-systolic) area measurements in pulsatile mode for each model. Refer to Supplementary Table S9 for underlying data.

Supplementary video file description: In all animated vector visualizations, cycle length was stretched from 1 s to 2 s to better display complex patterns, and the video file was exported with 24 fps.

S4 4D-flow MRI velocity vector visualizations in three TBAD models. Cycle length was slowed down to 2 s.

S5 CFD-FSI simulations displaying velocity vectors of fluid domain in three TBAD models.

S6 Entry tear close-up view of velocity vector visualizations of 4D-flow MRI and CFD-FSI simulations.

S7 Entry tear close-up view of velocity vector visualizations of 4D-flow MRI and CFD-FSI simulations. Identical data as in video S6, but with camera view rotated around aorta long axis.

S8 Exit tear close-up view of velocity vector visualizations of 4D-flow MRI and CFD-FSI simulations.
